# Physiological effects of a novel artificially synthesized antimalarial cyclic peptide: Mahafacyclin B

**DOI:** 10.1371/journal.pone.0188415

**Published:** 2017-11-30

**Authors:** Yuko Fujita, Panpaki Seekaki, Norichika Ogata, Kazuhiro Chiba

**Affiliations:** 1 Department of Applied Biological Science, Tokyo University of Agriculture and Technology, Fuchu, Tokyo, Japan; 2 Nihon BioData Corporation, Takatsu-ku, Kawasaki, Kanagawa, Japan; 3 Chitose Bio Evolution Pte. Ltd. The Central Singapore, Singapore; Hokkaido Daigaku, JAPAN

## Abstract

Mahafacyclin B is a cyclic peptide isolated from the latex of *Jatropha mahafalensis* and is an antimalarial agent. However, the physiological effects of mahafacyclin B in mammalian cells are not known. Here, we assessed the growth, morphology, and alterations in the transcriptome of CHO-K1 cells exposed to mahafacyclin B (0–22 μM). Mahafacyclin B at 2.2 μM did not affect the proliferation or death of CHO-K1 cells. Mahafacyclin B was not toxic to mammalian cells at 2.2 μM, which represents a normal physiological concentration at which mahafacyclin B retains its antimalarial properties. Interestingly, mahafacyclin B altered the size and morphology of CHO-K1 cells. Comparative transcriptomics revealed that mahafacyclin B modulated the expression of a specific subset of genes.

## Introduction

Cyclic peptides are polypeptide chains with a cyclic ring structure. Cyclic peptides have antibacterial, immunosuppressive, and antitumor activities [1]. Cyclic peptides, owing to their increased stability, high resistance to exo-peptidases and endo-peptidases, enhanced binding affinity, and selectivity for target biomolecules, are commonly investigated as therapeutic agents [2]. Biologically active cyclic peptides have been produced using biological and synthetic approaches, both of which have been adopted by pharmaceutical companies to develop novel medications. These novel medications represent a growing share of sales of all those produced worldwide [3].

However, using a biological approach to develop medications includes a high level of risk (e.g. contamination with the porcine circovirus in human vaccines) [4], and sometimes the biological manufacturing process can affect public safety and health [5]. Several therapeutic cyclic peptides are derived from biological processing, but new methods used to synthesize cyclic peptides are under development [6].

Previously, we established a method of chemically synthesizing the cyclic peptide mahafacyclin B, a molecule isolated from the latex of *Jatropha mahafalensis* [7–10]. We developed a soluble, tag-assisted peptide head-to-tail cyclization for this compound [11]. Newly synthesized cyclic peptides are requested not only for therapeutic intervention but also *in vitro* preclinical testing because biological extracts usually contain other molecules. For example, a biological extract and most lipopolysaccharide fractions are contaminated with peptidoglycans because both of them are components of bacterial membranes [12]. Therefore, by synthesising and testing synthetic products *in vitro*, it is possible to avoid the risk of unintended adverse effects. Using our method, >20 mg of cyclic peptide can be available.

Mahafacyclin B is a well-characterized antimalarial agent. Nevertheless, the physiological effects of mahafacyclin B in mammalian cells are not known [13]. Here, we examined the physiological effects of mahafacyclin B in Chinese hamster ovary-derived immortalized mammalian (CHO-K1) cells by measuring their growth, viability, and morphology. We also undertook gene-expression analyses of CHO-K1 cells exposed to mahafacyclin B. Determination of the appropriate concentration of drug to use is critically important for *in vitro* drug-exposure analyses in preclinical toxicology. High drug concentrations can induce radical transcriptome responses, whereas low concentrations can hamper determination of significant changes in gene expression. A previous study established a quantitative method to determine the appropriate drug concentrations that should be used for *in vitro* preclinical testing using Shannon’s information entropy and/or Kolmogorov complexity to assess transcriptomic changes [14, 15]. Comparing the amount of environmental change and the amount of transcriptomic change using phenobarbital in CHO-K1 cells, it was shown that Kolmogorov complexity is a better index of transcriptomic change than Shannon’s information entropy [16]. Here, we estimated Shannon's information entropy and the Kolmogorov complexity transcriptomic changes of CHO-K1 cells exposed to mahafacyclin B. It was suggested previously that a medium-sized molecule (e.g., cyclic peptide) can cause drastic regime shifting of gene-expression patterns based on the concentrations used. Here, we aimed to select the most appropriate concentration to analyze differently expressed genes, and found a specific subset of genes that were altered by exposure to mahafacyclin B.

## Results and discussion

### Synthesis of mahafacyclin B

**Fig 1:**
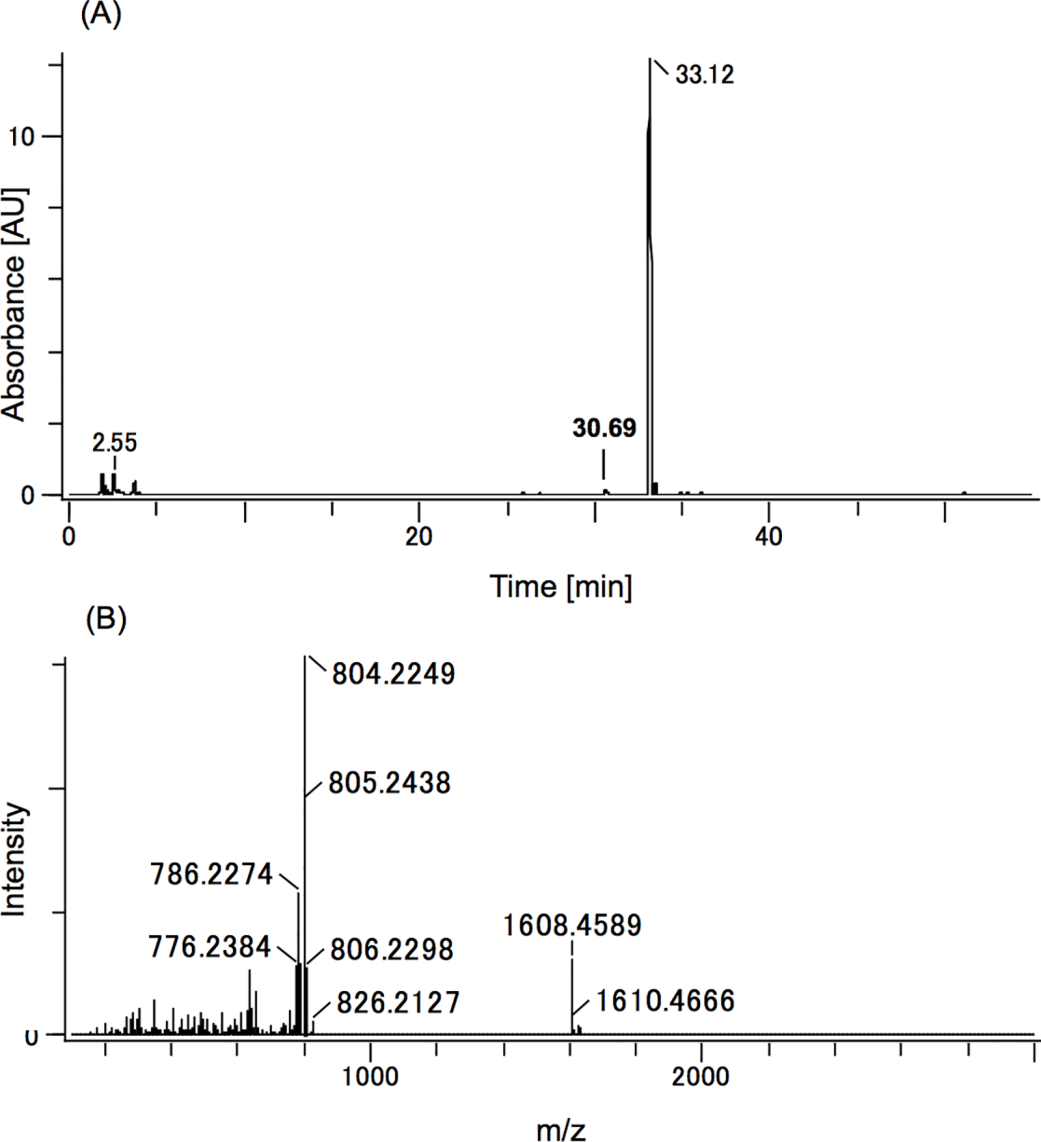
Purity of synthesized mahafacyclin B. (A) UV–Visible spectrum of LC-MS analysis of the sample (0%–100% 0.1%TFA CH_3_CN in 45 min). Peak purity was calculated using the peak area percentage. RT = 33.12 min was characterized as mahafacyclin B (96.8%) and RT = 30.69 min was found to be the epimerized variant (0.82%), the existence of which was determined by mass chromatogram (*m/z* 803–810, data not shown). (B) Mass spectrometry of the main peak in the UV–Visible spectrum of the LC-MS analysis of mahafacyclin B. *m/z* 804.2249 was characterized as [M+H], and *m/z* 1608.4589 (*m/z* 1607.4438 for the monoisotopic ion) as [2M+H].

We synthesized mahafacyclin B as reported previously [11]. After purification with semi-preparative reversed-phase high-performance liquid chromatography (HPLC), we finally obtained 20 mg of mahafacyclin B; (purity (UV) 96.8%, epimer <0.9%) (Fig 1). The retention time (RT) of this product was 33.12 min as assessed by HPLC. This RT was slightly longer than that of our product synthesized previously (RT = 31.43 min), but the ^1^H NMR spectrum of this product was similar to data published previously [8, 13].

### Mahafacyclin B dose not modulate the growth of CHO-K1 cells

CHO-K1 cells were exposed to mahafacyclin B. Freshly sub-cultured cells (0.8×10^6^/2 ml, viability 97.3723%) were cultured for 12 h in Dulbecco’s modified Eagle’s medium (DMEM)/F12 medium, and then cultured for 10 h in medium supplemented with 0, 0.22, 2.2, and 22 μM of the cyclic compound. Viable and dead cells were counted using a Vi-cell counter with Trypan Blue staining. In total, 5251 cells were analyzed.

**Fig 2:**
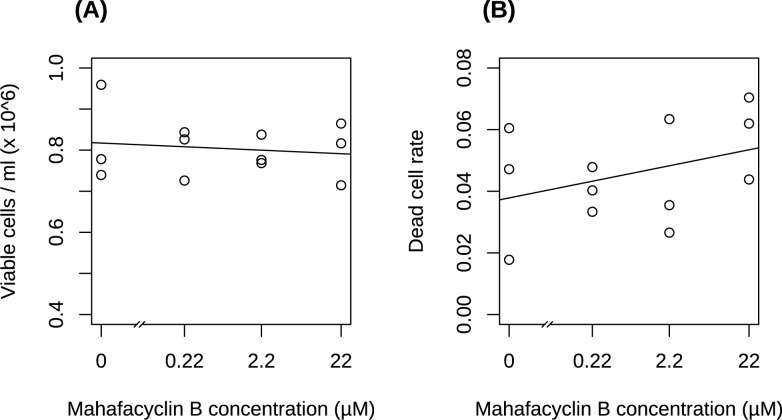
Mahafacyclin B does not affect proliferation of CHO-K1 cells. (A) Scatter plot of drug concentration *vs*. density of viable cells. (B) Scatter plot of drug concentration *vs*. dead cell rate. Cells were exposed to vehicle or mahafacyclin B for 10 h, and proliferation and viability were assessed using a cell counter and Trypan Blue.

Viable cell densities (in cells/ml (×10^6^)) were: 0.9590, 0.7780, and 0.7395 with 0 μM mafahacyclin B; 0.8261, 0.7260, and 0.8435 with 0.22 μM mahafacyclin B; 0.7761, 0.7684, and 0.8377 with 2.2 μM mahafacyclin B; 0.8647, 0.7145, and 0.8165 with 22 μM mahafacyclin B. There was no change in the number of viable cells after exposure to mahafacyclin B (Fig 2A). The slope of the regression line was not significantly different than 0 (p = 0.852). The value for the slope was −0.009495. The Akaike information criterion (AIC) of the linear model on the R function lm was −25.28037.

The dead cell rate was: 0.017751, 0.060465, and 0.047146 with 0 μM mahafacyclin B; 0.040268, 0.033333, and 0.047826 with 0.22 μM mahafacyclin B; 0.02657, 0.06338, and 0.035477 with 2.2 μM mahafacyclin B; 0.070393, 0.043814, and 0.061947 with 22 μM mahafacyclin B. There were differences in the rate of cell death in response to mahafacyclin B (Fig 2B). The slope of the regression line was significantly different than 0 (p = 0.00496). The value for the slope was −0.41055. The AIC of the linear model on the R function glm was 85.038.

Previously, cell viability was assessed by the 3-(4,5-dimethylthiazol-2-yl)-2,5-diphenyltetrazolium bromide (MTT) assay and the effects of cyclic peptides on cell viability were assessed using high concentrations (>50 μM) of each peptide [17]. In the present study, the effects of mahafacyclin B were apparent in terms of cell death, not cell viability. Previously, the antimalarial activity of mahafacyclin B was discovered using a concentration of 2.2 μg/ml [13]. However, cell growth was not inhibited with this concentration of mahafacyclin B.

### Mahafacyclin B modulates the morphology of CHO-K1 cells

Cellular morphology was assessed using a Vi-cell microscope and a phase-contrast microscope. Images were analyzed using CellProfiler [18]. We measured length of the major axis and minor axis, and the eccentricity of CHO-K1 cells in response to mahafacyclin B [19, 20].

**Fig 3:**
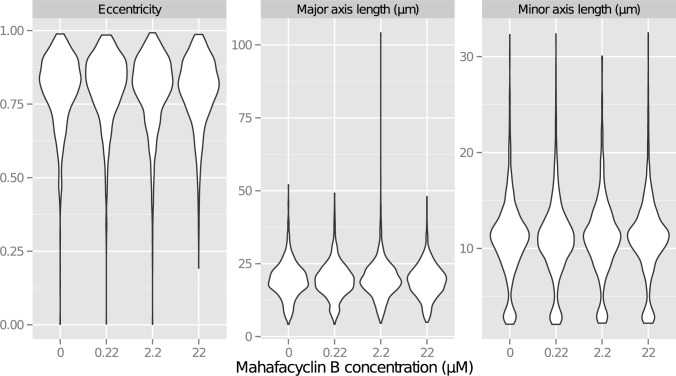
Mahafcyclin B alters the morphology of CHO-K1 cells. Morphology was measured using continuous boxplots for eccentricity, major-axis length, and minor-axis length of each cell in response to mahafacyclin B.

There were differences in the eccentricity of mahafacyclin B-induced cells (Fig 3). The slope of the regression line was significantly different than 0 (p = 0.0177). The value for the slope was −9.167e^−5^. The AIC of the linear model on the R function lm was −7721.999. The cell shape is thought to affect plasma membrane signaling [20]. In this context, the importance of the eccentricity had been predicted [20]. This eccentricity changing of mahafacyclin B-induced cells would change plasma membrane signaling and cause transcriptomic changes.

There were weak differences in the length of the major axis of mahafacyclin B-induced cells (Fig 3). The slope of the regression line was weakly significantly different than 0 (p = 0.0658). The value for the slope was −0.003683. The AIC of the linear model on the R function lm was 35952.98.

There were no differences in the length of the minor axis of mahafacyclin B-induced cells (Fig 3). The slope of the regression line was not significantly different than 0 (p = 0.56). The value for the slope was 7.807e^−4^. The AIC of the linear model on the R function lm was 31507.26.

### Mahafacyclin B modulates the expression of a subset of genes in CHO-K1 cells

We investigated the contribution of Shannon’s information entropy and Kolmogorov complexity to the transcriptomic changes of CHO-K1 cells by sequencing the transcriptomes of cells exposed to vehicle or mahafacyclin B. We measured the Shannon’s information entropy and the Kolmogorov complexity of those transcriptomes.

Shannon’s information entropies were: 11.79063, 11.71702, and 11.8423 with 0 μM mahafacyclin B; 11.92365, 11.82417, and 11.82417 with 0.22 μM mahafacyclin B; 11.51067, 11.84796, and 11.98672 with 2.2 μM mahafacyclin B; 11.79985, 11.79642, and 11.84944 with 22 μM mahafacyclin B. The slope of the regression line was not significantly different than 0 (p = 0.904). The value for the slope was −0.005269. The AIC of the model was −21.59212.

**Fig 4:**
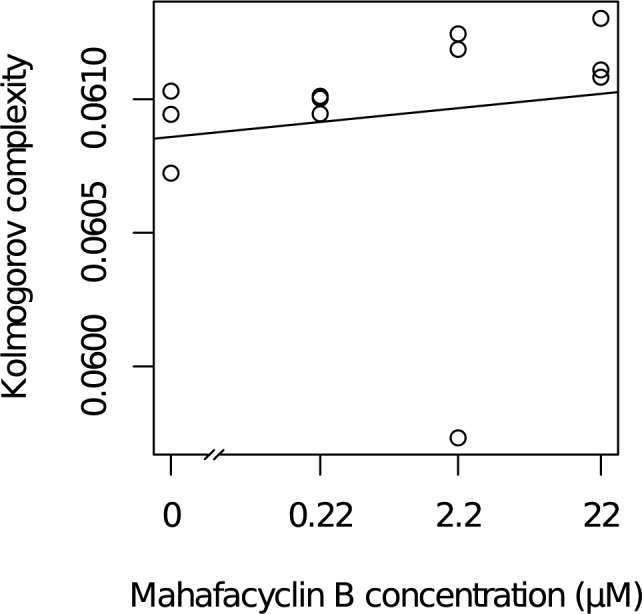
Comparison between transcriptomic and environmental changes in CHO-K1 cells treated with mahafacyclin B. Scatter plot of drug concentration *vs*. Kolmogorov complexity representing the transcriptome of CHO-K1 cells cultured for 10 h in DMEM/F12 medium supplemented with mahafacyclin B.

The Kolmogorov complexities were: 0.0607232, 0.0609433, and 0.0610302 with 0 μM mahafacyclin B; 0.0610032, 0.0609453, and 0.0610109 with 0.22 μM mahafacyclin B; 0.0597326, 0.0612445, and 0.0611866 with 2.2 μM mahafacyclin B; 0.0610824, 0.0611094, and 0.0613025 with 22 μM mahafacyclin B. With 2.2 μM of mahafacyclin B, we found an outlier; the transcriptomic data showed extremely low values for Kolmogorov complexity. We, therefore, removed the transcriptomic data from this sample before further analyses. The slope of the regression line was significantly different than 0 (p = 0.0288) (Fig 4). The value for the slope was 2.238e^−4^. The AIC of the model was −134.0088. The AIC of the model which utilized Kolmogorov complexity was lower compared with that utilizing Shannon’s information entropy. Kolmogorov complexity was a better index of transcriptomic data than Shannon’s information entropy. The AIC (Akaike's information criterion) is an index for selecting statistical models. To increase the numbers of parameters of statistical models makes models fit better to the data and phenomenon. However, fitting model having too much model is useless. The AIC solves this problem. We chose the model having the lowest AIC. The Shannon's information entropy is an index of data. The transcriptome which a few genes occupy major region represents small information entropy. The transcriptome which many genes occupy major region represents large information entropy. Therefore, the Shannon's information entropy was used as a quantitative marker of cellular differentiation/dedifferentiation [14]. The Kolmogorov complexity is also an index of data. The elementary theories of Shannon's information and Kolmogorov complexity have a common purpose. Kolmogorov complexity is the minimum number of bits from which a particular message or file can effectively be reconstructed [15].

Studies comparing Shannon’s entropy and Kolmogorov complexity have shown that Kolmogorov complexity is, in fact, a better indicator of regime shifting of transcriptomic data than Shannon’s information entropy. In one study, cells cultured with drug concentrations lower than the tipping point showed uniformly high Kolmogorov complexity, whereas those cultured with concentrations higher than the tipping point showed uniformly low Kolmogorov complexity using primary cultured tissues and phenobarbital [14, 15]. In another study, cells cultured with low drug concentrations showed low Kolmogorov complexity, whereas those cultured with higher drug concentrations showed low Kolmogorov complexity using mammalian cell lines and phenobarbital [16]. In primary cultured cells and cell lines, the relationship between drug concentration and Kolmogorov complexity was reversed. In the present study, cells cultured with low drug concentrations showed low Kolmogorov complexity and those cultured with high drug concentrations showed low Kolmogorov complexity. Increased Kolmogorov complexity with increasing drug concentration is observed in cell lines. Changes in the Kolmogorov complexity indicate drastic regime shifting of genomic expression systems. Hence, a medium-sized molecule (e.g., cyclic peptide) can cause drastic regime shifting of genomic expression systems in a similar manner to that of small molecules.

Using traditional RNA-sequencing methods with a heterogeneous population of cells can induce variance in RNA-sequencing data pools. Treatment with phenobarbital and mahafacyclin B could increase the heterogeneity of immortalized cell lines. To address this possibility, we analyzed the morphological heterogeneity of CHO-K1 cells in response to mahafacyclin B using Shannon’s information entropy. Shannon’s information entropy for morphological eccentricity was compared with the concentration of mahafacyclin B. The use and interpretation of Shannon’s information entropy as a measure of diversity and homogeneity is justified by mathematically founded arguments [21]. The slope of the regression line was not significantly different than 0 (p = 0.673). Shannon’s information entropy for major-axis length was compared with the concentration of mahafacyclin B. The slope of the regression line was not significantly different than 0 (p = 0.192). Shannon’s information entropy for minor-axis length was compared with the concentration of mahafacyclin B. The slope of the regression line was not significantly different than 0 (p = 0.366). These results indicate that increased cellular heterogeneity does not explain the observed phenomenon: increasing Kolmogorov complexity is correlated with increasing drug concentration.

### Identification of genes modulated by mahafacyclin B

**Fig 5:**
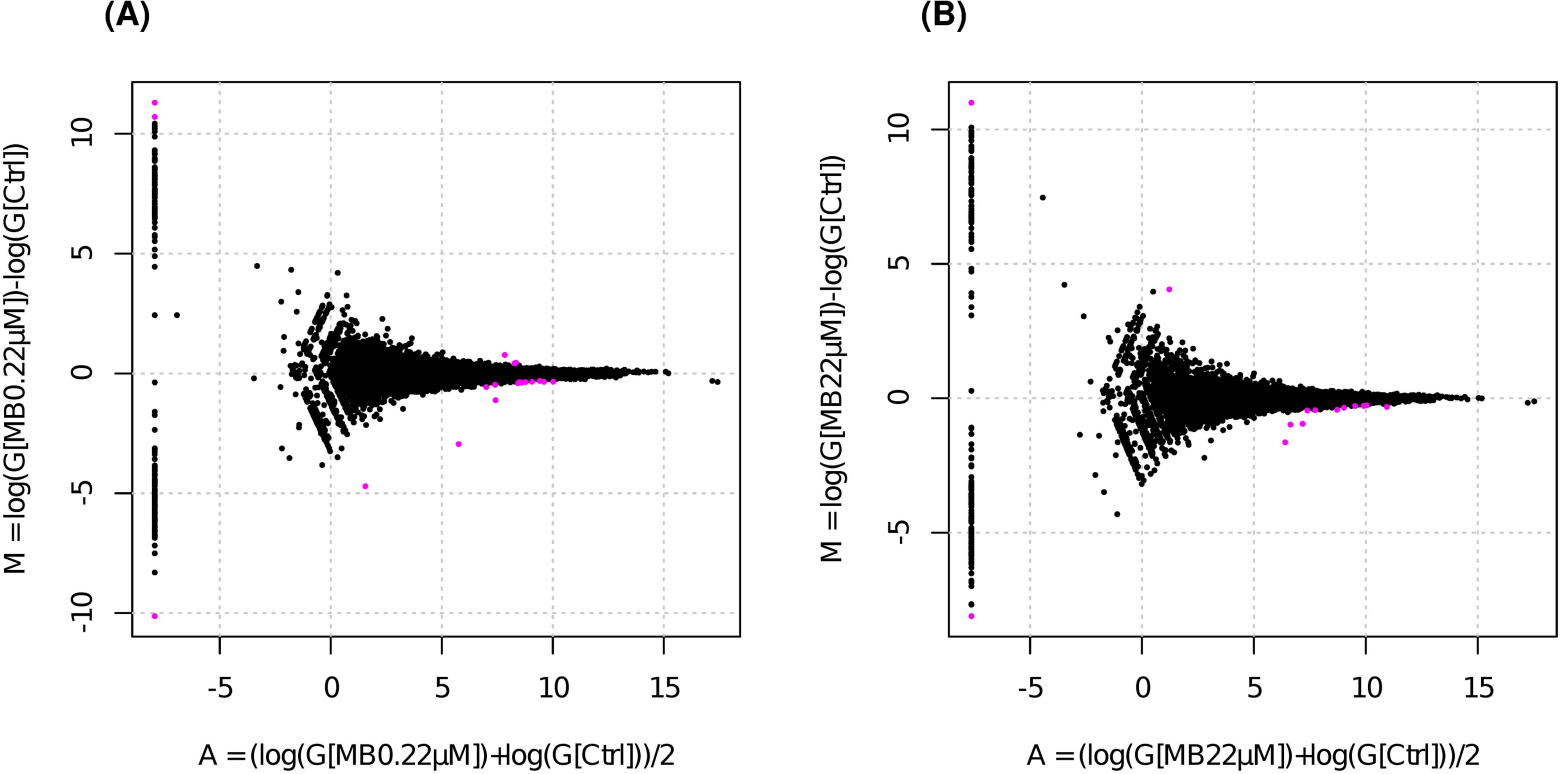
Mahafacyclin B alters the expression of a subset of genes in CHO-K1 cells. Genes marked in purple were detected as differentially expressed at the 5% false-discovery rate (FDR) using TCC. (A) Scatter plot of differentially expressed genes in response to 0 or 0.22 μM mahafacyclin B. (B) Scatter plot of differentially expressed genes in response to 0 or 22 μM mahafacyclin B.

To investigate the effects of mahafacyclin B on gene expression, we undertook two comparisons of transcriptomic changes that occurred in response to mahafacyclin B (0 *vs*. 0.22 μM, 0 *vs*. 22 μM). We obtained 19 statistically significant differentially expressed genes (false discovery rate (FDR) < 0.05) using data from cells exposed to a low concentration of mahafacyclin B (0 *vs*. 0.22 μM, Fig 5A, Table 1) and 14 from data used with a high concentration of the cyclic peptide (0 *vs*. 22 μM, Fig 5B, Table 2). Six genes were found to overlap in both comparisons, and these were identified as LOC100754005, LOC100689425, LOC103164497, LOC100752965, Kmt2a, and Ino80d. Among these genes, LOC103164497 (NCBI Reference Sequence: XP_007652779.1) expression was increased in response to mahafacyclin B, but its function is not known. However, XP_007652779.1 shared sequence similarity with XM_007643794.2, a predicted protein similar to the *Cricetulus griseus* retrovirus-related Pol polyprotein from transposon 17.6-like (LOC103163056) (eValue, 0.0; identities, 756/758 (99%); gaps 0/758 (0%): tblastn). Pol polyprotein was predicted to have aspartic-type endopeptidase activity, and could be used to degrade mahafacyclin B intracellularly. Pol polyprotein was also predicted to be involved in viral entry into host cells. Endogenous retroviral sequences are present in high copy numbers within the genomes of all species, whereas endogenous production of retroviruses can be induced chemically [22–24]. Therefore, mahafacyclin B could be used (in theory) to discover novel retroviruses. Expression of chromatin-remodeling genes (Kmt2a, Ino80d [25], Ash1l [26], and BOX [27]) decreased in response to mahafacyclin B, indicating that epigenetic inheritance could occur to prolong the effects of the peptide. Reduced expression of genes related to cell division (ORC1-like [28], Cdk6, PRC1 [29], RPA1, and Cdk11B) indicate that cell-cycle progression was slowed. However, we did not observe a reduction in cell number in response to mahafacyclin B. Expression of genes related to regulation of ubiquitin-mediated proteolysis (Wdfy3 [30], FBXW-2 like: LOC100754385 [31]) decreased. Interestingly, expression of genes related to ATP synthesis (LOC100762406:ATP5E, LOC100767351:COX7A2) increased in response to mahafacyclin B. The CHO cell line has been used for protein production. Recently, it was shown that ATP has the properties of a biological hydrotrope [32]. Therefore, mahafacyclin B may increase ATP concentrations intracellularly and improve protein-folding efficiency. Reduced expression of a gene encoding a structural component of microtubules (Tubb) and a gene related to the regulation of cell–cell junctions (Heg1 [33]) could also be associated with morphological changes in response to high concentrations of mahafacyclin B. No apoptotic genes were induced by mahafacyclin B.

**Table 1:** Characterization of differentially expressed genes in response to 0 or 0.22 μM mahafacyclin B.

genenames	ctrl_1	ctrl_2	ctrl_3	mB0022_1	mB0022_2	mB0022_3	a.value	m.value	p.value	FDR
LOC100754005	100.72	213.25	147.08	0	0	53.02	5.76068679	-2.948403767	7.43e^-59^	1.16e^-54^
LOC100689425	318.42	259.97	177.53	33.48	178.63	131.83	7.419020938	-1.118562591	2.66e^-21^	2.07e^-17^
LOC100754385	0.06	0	58.93	0	0	0	-7.927587797	-10.12573379	2.51e^-15^	1.31e^-11^
Orc1	82.58	215.03	230.47	393.52	275.37	227.17	7.835309472	0.770332244	3.54e^-13^	1.38e^-9^
LOC103159045	28.19	0	14.04	0	1.81	0	1.557115936	-4.707881333	6.63e^-09^	2.07e^-5^
LOC100750843	0	0	0	0	27.97	0	-7.927587797	11.2964614	1.40e^-7^	0.000364688
Kmt2a	1035	1421	1076	1070	776	889	10.01673187	-0.34392997	7.89e^-7^	0.001758723
Cdk6	852	1064	738	749	581	709	9.600659774	-0.350428827	1.54e^-6^	0.003013339
LOC100762406	245.12	311.36	277.07	370.04	433.87	328.09	8.338802637	0.448697588	1.91e-6	0.003309558
Zbed6	348	515	346	321	255	312	8.43084409	-0.407949544	4.32e^-6^	0.006738624
LOC103158912	131.36	221.31	125.67	123.64	98.81	91.99	7.001583951	-0.568907307	7.69e^-6^	0.010901861
Wdfy3	368	571	418	396	272	350	8.613277583	-0.38067373	9.47e^-6^	0.012311282
Psd3	565	716	540	499	457	453	9.060777063	-0.348847864	1.89e^-5^	0.022709501
LOC100752965	469.28	580.54	444.18	424.57	336.73	382.02	8.769469382	-0.362967358	2.38e^-5^	0.026497281
Ino80d	160.68	266.94	178	167.03	124	134.97	7.397011542	-0.47321388	3.08e^-5^	0.032004724
LOC100767351	282.85	389.85	148.34	315.02	485.57	273.3	8.268980549	0.419540907	3.42e^-5^	0.033339144
Lnpep	350	533	361	337	287	322	8.495561815	-0.359744531	3.86e-5	0.033986166
LOC103164497	0	0	0	18.28	0	0	-7.927587797	10.70501347	3.97e^-5^	0.033986166
Ash1l	643	919	730	651	573	592	9.4095832	-0.312163717	4.14e^-5^	0.033986166

**Table 2:** Characterization of differentially expressed genes in response to 0 or 22 μM mahafacyclin B.

genenames	ctrl_1	ctrl_2	ctrl_3	mB2200_1	mB2200_2	mB2200_3	a.value	m.value	p.value	FDR
Abca1	729	987	772	657	584	712	9.521511425	-2.90e^-1^	3.91e^-5^	0.047058609
Bbx	568	675	547	488	372	488	9.02125406	-3.51e^-1^	5.75e^-6^	0.011253935
Heg1	1011	1198	1012	865	671	1011	9.902945621	-2.87e^-1^	2.73e^-5^	0.035568464
Ino80d	160.68	266.94	178	147.55	113.13	159	7.387755871	-4.55e^-1^	2.55e^-5^	0.035568464
Kmt2a	1035	1421	1076	968	634	1215	10.03786279	-2.65e^-1^	4.37e^-5^	0.048818452
LOC100689088	260.13	127.9	206.61	0	0	319.15	7.177458179	-9.51e^-1^	1.50e^-12^	1.17e^-8^
LOC100689425	318.42	259.97	177.53	172.76	258.89	106.46	7.737809016	-4.44e^-1^	2.37e^-5^	0.035568464
LOC100752965	469.28	580.54	444.18	363.65	299.46	402.43	8.71777029	-4.30e^-1^	1.70e^-7^	0.000532847
LOC100754005	100.72	213.25	147.08	33.9	0	108.28	6.396727017	-1.64e^0^	6.63e^-28^	1.04e^-23^
LOC100758733	22.73	0	0.56	0	0	0	-7.621132898	-8.10e^0^	2.22e^-6^	0.005787151
LOC100761053	93.12	158.27	172.19	67.21	79.87	60.63	6.632165935	-9.81e	5.01e^-12^	2.61e^-8^
LOC100761345	0	0	1.6	7	11.75	9.35	1.219005141	4.05e^0^	8.14e^-6^	0.014158601
LOC103164155	2070	2566	2060	1757	1321	2079	10.93807586	-3.21e^-1^	1.06e^-7^	0.000416302
LOC103164497	0	0	0	0	21.69	0	-7.621132898	1.10e	4.07e^-6^	0.00909298

## Conclusions

We characterized the physiological effects of the antimalarial agent mahafacyclin B. Exposure to mahafacyclin B had no effect on proliferation but, at certain concentrations, induced cell death. Mahafacyclin B is, therefore, not toxic at 2.2 μM, which is a physiologically relevant concentration. We found that mahafacyclin B altered the expression of a specific subset of genes; no apoptotic genes were induced by mahafacyclin B. The increase of peptidase enzyme was not observed in this study. These findings also indicated the safety and stability of mahafacyclin B as the antimalarial drug. Mahafacyclin B induced expression of ATP synthesis genes; mahafacyclin B could be used for an industrial protein production enhancement not only an antimalarial agent. Kolmogorov complexity was a better index than Shannon’s information entropy in assessing the comparative transcriptomics of CHO-K1 cells exposed to mahafacyclin B.

## Materials and methods

### Synthesis of mahafacyclin B

All reagents and solvents for the synthesis of mahafacyclin B were purchased from Watanabe Chemicals (Hiroshima, Japan), Kanto Chemicals (Tokyo, Japan), Tokyo Kasei Kogyo (Tokyo, Japan), Sigma–Aldrich (Saint Louis, MO, USA), Jitsubo (Tokyo, Japan), and Wako Pure Chemical Industries (Osaka, Japan).

We synthesized mahafacyclin B as reported previously [11]. Briefly, 613.4 mg (0.33 mmol) of the hydrophobically tagged heptapeptide Fmoc-Phe-Phe-Gly-Thr(tBu)-Phe-Phe-(TAG)-Gly-OMe was treated with 2% 1,8-diazabicyclo[5.4.0]undec-7-ene and piperidine in tetrahydrofuran (THF; 16.5 ml) for 5 min and hydrolyzed with 1 M aqueous LiOH in THF (16.5 ml) for 4 h at room temperature.

The cyclization step was accomplished with 1.2 equivalents of (1-[bis(dimethylamino)methylene]-1H-1,2,3-triazolo[4,5-b]pyridinium 3-oxid hexafluorophosphate) (HATU) and 1-hydroxy-7-azabenzotriazole (HOAt) and 2.4 equivalents of *N*,*N*-diisopropylethylamine (DIPEA) in 10% dimethylformamide (DMF) in dehydrated THF (16.5 ml; 20 mM) at 40°C for 1 h. Global deprotection of hydrophobically tagged cyclic peptide was conducted with 2.5% triisopropylsilane (TIS) and H_2_O in trifluoroacetic acid (TFA; 10 ml) for 4 h at room temperature.

The reaction mixture was filtered with Celite™ 454, and DIPE was added to the evaporated filtrate for product precipitation. The precipitability of the product was decreased significantly as the volume of TFA for washing the filter increased (filtrate 40 ml after evaporation), so the supernatant was centrifuged three times at 3000 rpm for 10 min at −9°C. After the precipitate was dissolved in CH_3_Cl/MeOH, evaporated, and dried, 75.0 mg of crude product was obtained. The purity of the desired product (as assessed by HPLC) was decreased to 62%, thereby increasing the rate of epimer formation during alkaline hydrolysis.

### Induction assay

All chemicals used in the induction assay were of analytical grade. Mahafacyclin B was dissolved in solvent (water and acetonitrile, 1:1) to make a stock solution, which was diluted further and added to the culture medium to yield final concentrations of 0.22, 2.2, and 22 μM. CHO-K1 growth medium was replaced with mahafacyclin B-containing medium before all induction assays. For each experiment, CHO-K1 cells (American Type Culture Collection, Manassas, VA, USA) were cultured with mahafacyclin B or vehicle for 10 h. Cells were counted using a Vi-cell counter (Beckman Coulter, Brea, CA, USA). CHO-K1 cells were exposed to mahafacyclin B. Freshly sub-cultured cells (0.8×10^6^/2 ml, viability = 97.37%) were cultured for 12 h in DMEM/F12 medium, and then cultured for 10 h in medium supplemented with 0, 0.22, 2.2, and 22 μM of the cyclic compound. Viable and dead cells were counted using a Vi-cell counter with Trypan Blue staining. In total, 5251 cells were analyzed.

All data were processed using bash v3.2 and visualized using R. The percentage of viable and dead cells was analyzed using R (R project for statistical computing, Vienna, Austria) and Fitting Linear Models (lm). The number of viable cells, the eccentricity of cells, the length of the major axis of cells, the length of the minor axis length of cells, the Shannon’s information entropies of transcriptomes and the Kolmogorov complexities of transcriptomes in response to mahafacyclin B were analyzed using simple linear regression analyses. The simple linear regression analyses were performed using R. The rate of cell death in response to mahafacyclin B was analyzed using generalized linear models (glm).

Induction cultures were terminated using a QIAshredder; (Qiagen, Hilden, Germany) and the cells were kept at −80°C before analyses. RNA was extracted using an RNeasy mini kit (Qiagen). mRNA libraries were prepared for RNA sequencing using a TruSeq RNA Sample kit (Illumina, San Diego, CA, USA) per manufacturer protocols. These libraries were sequenced using a NextSeq 500 sequencer (Illumina) per manufacturer protocols. Short-read data were deposited in the Short Read Archive (project ID, DRA005920) of the DNA Data Bank of Japan. All raw sequencing reads were mapped to the CHO-K1 RefSeq assembly (ID GCF_000223135.1) using rsem [34].

Kolmogorov complexity was measured as described previously [15] and analyzed using R and lm. Analyses of differentially expressed genes were done using R and the TCC v1.1.99 [35]. We estimated library normalization factors using TbT method with TCC package. Statistically significant differentially expressed genes were extracted with false discovery rate (FDR).
